# Polar Constituents and Biological Activity of the Berry-Like Fruits from *Hypericum androsaemum* L.

**DOI:** 10.3389/fpls.2016.00232

**Published:** 2016-03-01

**Authors:** Giovanni Caprioli, Alessia Alunno, Daniela Beghelli, Armandodoriano Bianco, Massimo Bramucci, Claudio Frezza, Romilde Iannarelli, Fabrizio Papa, Luana Quassinti, Gianni Sagratini, Bruno Tirillini, Alessandro Venditti, Sauro Vittori, Filippo Maggi

**Affiliations:** ^1^School of Pharmacy, University of CamerinoCamerino, Italy; ^2^Rheumatology Unit, Department of Medicine, University of PerugiaPerugia, Italy; ^3^School of Bioscience and Veterinary Medicine, University of CamerinoCamerino, Italy; ^4^Department of Chemistry, Sapienza University of RomeRome, Italy; ^5^Department of Environmental Biology, Sapienza University of RomeRome, Italy; ^6^School of Science and Technology, University of CamerinoCamerino, Italy; ^7^Department of Biomolecular Sciences, University of UrbinoUrbino, Italy

**Keywords:** *Hypericum androsaemum*, berry-like fruits, phytochemicals, antioxidant, cytotoxicity, immunomodulatory

## Abstract

*Hypericum androsaemum*, also known as Tutsan, is a small evergreen shrub common in the Mediterranean basin where it is traditionally used as diuretic and hepatoprotective herbal drug. This plant possesses the peculiarity to produce fleshy and berry-like fruits that ripen from red to shiny black. In the present work, the chemical constituents of methanolic extracts and infusions of red and black fruits were analyzed by HPLC, and correlated with their antioxidant properties which were evaluated by the DPPH, β-Carotene/linoleic acid, and hypochlorous acid tests. In addition, the red pigment of the fruit was isolated by column chromatography and structurally elucidated by NMR. Results showed that *H. androsaemum* fruits contain high amounts of shikimic and chlorogenic acids, while their color was given by a tetraoxygenated-type xanthone, reported for the first time in *Hypericum* species. The red berries infusion gave the highest content of total phenolic compounds, DPPH, and hypochlorous acid scavenging activity, and β-carotene bleaching. Cytotoxicity of the berries extracts on three human tumor cell lines (malignant melanoma, breast adenocarcinoma, and colon carcinoma) was evaluated by MTT assay, and relevant inhibition on colon carcinoma cells (IC_50_ value of 8.4 μg/mL) was found. Finally, the effects of red berries extract on the immune system were evaluated by peripheral blood mononuclear cell (PBMC) proliferation assay that revealed a strong stimulation on lymphocytes at low doses (0.4–6 μg/mL).

## Introduction

*H. androsaemum* [Hypericaceae, sect. *Androsaemum* (Duhamel) Godr.], well known as “tutsan,” is a small evergreen shrub fairly common in damp woods and hedgerows (100–1400 m of altitude) within the Mediterranean Basin, mainly in Western and Southern Europe, South-Western Asia, North Africa, while it has been introduced elsewhere (Allen and Hatfield, 2004). The plant possesses big, opposite, sessile, ovate and slightly aromatic leaves bearing translucent glands, but lacking black nodules which are the secretory structures storing naphtodiantrones (Perrone et al., 2013). The flowers are large, yellow, with small clearer spots on the petals. Among *Hypericum* species, the plant has the peculiarity to produce capsules that are not dry at ripening, but become more or less fleshy and berry-like. The ease of cultivation of the plant and the natural color variation in the fresh capsules has been exploited through directed breeding and line selection, resulting in economically highly successful cultivars.

*H. androsaemum* is used as an important traditional medicine in Europe. For instance, in Portugal its leaves are used as diuretic and to treat liver, kidney and bladder ailments (Valentão et al., 2002). In England, they are mixed with lard to produce an ointment for dressing cuts and wounds (Phillips, 1977; Allen and Hatfield, 2004).

So far, much of the phytochemical and pharmacological studies focused on leaves, revealing the presence of several flavonoids and phenolic acids, mainly chlorogenic acids, and quercetin derivatives which are responsible for the plant hepatoprotective properties (Valentão et al., 2002, 2004). However, scientific works on the showy tutsan's berries were not yet provided. Interestingly, the pigment giving the reddish-black color to the capsular tissue has not yet been identified.

Searching for new fruits with healthy properties, in the present work we reported a comprehensive analysis on the polar constituents and biological activities, namely the antioxidant power, cytotoxicity on tumor cells, and immunomodulatory capacities of *H. androsaemum* berries collected from spontaneous and cultivated plants in central Italy. On the above, we identified the balsamic period during which the berries are richer in active constituents.

## Materials and methods

### Plant material

Fleshy red and black capsules of *H. androsaemum* were collected in July–August 2014 from cultivated and wild-growing plants from different localities of central Italy belonging to Emilia-Romagna, Marche, and Abruzzo regions (Table 1). Voucher specimens were authenticated and deposited in the *Herbarium Universitatis Camerinensis* (CAME, included in the online edition of Index Herbariorum c/o School of Biosciences and Veterinary Medicine, University of Camerino, Italy), and archived in the anArchive system for botanical data (anArchive system, http://www.anarchive.it).

**Table 1:** **Main information on the investigated “berry-like” fruits of ***Hypericum androsaemum*****.

**Color**	**Sample N**.	**Region**	**Collection site**	**GPS coordinates**	**Altitude (m a.s.l.)**	**Voucher number^*a*^**	**Habitat**
Red	1	Emilia Romagna	Il Giardino delle Erbe (Casola Valsenio)	N 44°13′48″; E 11°37′26″	265	CAME 26763	Cultivated
	2	Marche	Torrone (Camerino)	N 43°15′72″; E 13°10′36″	675	CAME 26708	*Castanea sativa* old coppice
	3	Marche	Paganico (Camerino)	N 43°07′38″; E 13°06′30	620	CAME 26934	*Castanea sativa* old coppice
Black	4	Marche	University Botanical Garden (Camerino)	N 43°08′06″; E 13°04′09″	638	CAME 26757	Cultivated
	5	Marche	Torrone (Camerino)	N 43°15′72″; E 13°10′36″	675	CAME 26708	*Castanea sativa* old coppice and conifers reforestation
	6	Marche	Gorgovivo (Serra San Quirico)	N 43°26′01″; E 13°01′05″	160	CAME 26754	*Orno-ostryetum* s.l. old coppice
	7	Abruzzo	Monte Morrone (Pratola Peligna)	N 42°07′16″; E 13°55′05″	1005	CAME 26755	Between *Quercus cerris* forest and conifers reforestation

a *CAME, Herbarium Universitatis Camerinensis, School of Biosciences and Veterinary Medicine, Sect. of Botany and Ecology, University of Camerino (Italy)*.

### Reagents and standards

The analytical standards of polyphenols, hypericin, hyperforin, and ascorbic acid were purchased from Sigma-Aldrich (Milano, Italy) and individual stock solutions and standard working solutions were prepared in methanol. HPLC-grade methanol, acetonitrile, acetone, ethyl acetate (≥99.9%) and phosphoric acid were purchased from Sigma-Aldrich (Milano, Italy). HPLC-grade formic acid was supplied by Merck (Darmstadt, Germany). All solvents and solutions were filtered through 0.45-μm PTFE filters purchased from Phenomenex (Bologna, Italy). For antioxidant assays, 1,1-diphenyl-2-picrylhydrazyl radical (DPPH), butylated hydroxytoluene (BHT), 6-hydroxy-2,5,7,8-tetramethylchroman-2-carboxylic acid (Trolox), Tween 20, β-carotene, linoleic acid, sodium hypochlorite, taurine, phosphate buffer saline (PBS), and potassium iodide were purchased from Sigma-Aldrich-Fluka (Milan, Italy). Potassium hydroxide, hexane and ethanol were purchased from Sigma–Aldrich (Milan, Italy). Supelco 37 Component FAME Mix was purchased from Supelco (Bellefonte, PA, USA). Anhydrous sodium sulfate was purchased from Fluka-Riedel-deHaën (Milano, Italy) and methanol from Panreac Quimica SA (Barcelona, Spain). Deionized water (>18 MΩ cm resistivity) was obtained from a Milli-Q SP Reagent Water System (Millipore, Bedford, MA, USA).

### Preparation of extracts and infusions

The fresh fruits of *H. androsaemum* were grinded using liquid nitrogen. The finely powdered material (500 mg) was extracted with 5 mL of methanol by sonication (60 min, at ambient temperature). After centrifugation at 5000 rpm for 10 min, the extracts were transferred to volumetric flask, which was then filled up to 5 mL with extraction solvent. The sample solutions were filtered through a 0.45 μm pore size nylon membrane filter (Phenex, Phenomenex, Torrance, CA, USA) before injection into HPLC-DAD. All samples were stored in a refrigerator at the temperature of 4°C until analysis. Each sample was analyzed in triplicate. For tea preparation (water infusion), 5 g of fresh red and black capsules were treated with boiling water (100 mL), and infused for 15 min. The obtained liquid was then filtered and cooled. The volume was adjusted to 100 mL in a volumetric flask. An aliquot of the infusion was then filtered through a 0.45 μm membrane and used for HPLC analysis. Each sample was analyzed in triplicate.

### HPLC-DAD analysis

#### Analysis of polyphenols

HPLC-DAD studies were performed using a Hewlett-Packard HP-1090 Series II (Palo Alto, CA, USA), equipped with a vacuum degasser, a binary pump, an autosampler and a model 1046A HP photodiode array detector (DAD). Chromatographic separation was accomplished on a Synergi Polar-RP C18 (4.6 × 150 mm, 4 μm) analytical column from Phenomenex (Chesire, UK). The column was preceded by a security cartridge. The mobile phase for HPLC-DAD (diode array detector) analyses was a mixture of (A) water with 0.1% formic acid (v/v) and (B) methanol, flowing at 0.7 mL/min in isocratic conditions: 60% A, 40% B. The injection volume was 5 μL. UV spectra were recorded in the range 210–350 nm for 11 compounds, where 210 nm was used for quantification of shikimic acid, gallic acid, (+)-catechin hydrate, (-)-epicatechin; 310 nm for *p*-coumaric acid, and *trans*-resveratrol; 325 for caffeic acid and trans-ferulic acid, 3-O-caffeoylquinic acid, 5-O-caffeoylquinic acid, and 3,5-di-O-caffeoylquinic acid.

#### Analysis of flavonoids, hypericin, and hyperforin

Each extract was chromatographed in reverse phase by an HPLC HP 1090 equipped with an autosampler HP series 1090, ternary pump and DAD detector, following a method previously developed in our research group (Zorzetto et al., 2015). UV/Vis spectra were recorded in the range 210–650 nm, where 210 nm was used for quantification of rutin, hyperoside, isoquercitrin, quercetin, and quercitrin, 270 nm for hyperforin, and 590 nm for hypericin.

#### Evaluation of anthocyanins and betacyanins occurrence

The freeze-dried powder (1 g) of *H. androsaemum* fruits (sample 4) was extracted at room temperature under stirring, twice with 30 mL of 70% EtOH adjusted to pH 2.0 by HCOOH, over 1 h as total time. The supernatant was filtered, dried under vacuum, and redissolved with 5–10 mL (exactly measured) of the following mixture: acidic water/acetonitrile/methanol 8:1:1, final pH 2.0, giving a red-brownish color. Sample was then directly analyzed by HPLC/DAD (Mulinacci et al., 2008), but the analyses of the chromatographic profile at 520 nm, the wavelength of choice for monitoring of anthocyanins excluded the presence of this class of compounds. Furthermore, an extraction was carried out with ethanol/water 60:40 on the lyophilized sample; the extract obtained was red-brownish, but once again the analyses in HPLC/DAD excluded the presence of betacyanins.

### Method validation

#### Polyphenols

Calibration curves of the 11 analyzed compounds (linear range 0.5–100 mg/L), correlation coefficient and LODs and LOQs are reported in Table S1. The obtained recoveries for all compounds, evaluated spiking the samples at two different level of concentration (10 and 50 mg/L) with a standard mixture of the 11 compounds, were in the range 92–97 and 99–102%, respectively, with a % RSDs <12% (*n* = 5) in all cases. Retention time stability was utilized to demonstrate the specificity of the HPLC-DAD method. Reproducibility of the chromatographic retention time for each compound was examined five times per day over a 5-day period (*n* = 25). The retention times using this method were stable with a percent RSD value of ≤1.89%.

#### Flavonoids, hypericin, and hyperforin

Calibration curves of the eight analyzed compounds (linear range 5–100 mg/l), correlation coefficient, LODs, and LOQs are reported in Table S1. Retention time stability was used to demonstrate the specificity of the HPLC-DAD method. Reproducibility of the chromatographic retention time for each compound was examined five times per day over a 5-day period (*n* = 25). The retention times using this method were stable with a percent RSD value of ≤2.52%.

#### Estimation of the total amount of phenolic compounds and flavonoids

A modified method of Folin-Ciocalteu, according to Singleton and Rossi (1965) was used for determination of the total amount of phenolics. Seven milliliter of distilled water, 0.5 mL of Folin-Ciocalteu reagent and 0.5 mL of extract (or standard solution of gallic acid) were mixed. After 3 min, 2 mL of 20% Na_2_CO_3_ were added and incubated in darkness at room temperature for 90 min. The absorbance was measured at 685 nm and the results expressed in mg of gallic acid/kg fresh fruit. All measures were repeated three times and averaged. The flavonoids content was estimated by the AlCl_3_ method (Lamaison and Carnat, 1990). One mL of methanol extract solution was added to 1 mL of 2% methanolic AlCl_3_ 6H_2_O. The absorbance was measured 10 min later at 430 nm comparatively to a rutin standard. The results were expressed as mg rutin/kg fresh fruit. All measures were repeated three times and the results were averaged.

#### Isolation and structural elucidation of the fruit pigment

The red berries (20.0 g, sample 2) were exhaustively extracted with ethanol 96% (3 × 250 mL) for 48 h and the extracts were gathered altogether and concentrated under reduced pressure obtaining an aqueous sunspension which was further freeze-dried to recover finally 3.0 g of crude extract. A portion of the crude extract (2.0 g) was subjected to a first column chromatography (CC) on silica gel (60.0 g) using *n*-butanol saturated with water (BuOH/H_2_O 82:18 v/v) as eluting system. From this chromatographic run, five compounds were directly identified, i.e., 1,2,3,5-tetrahydroxyxanthone [Fr. 3] (3.9 mg; Trong Tuan et al., 2012), isoquercitrin (Manguro et al., 2003; Han et al., 2004), and 7-*O*-glucosyl luteolin (Lu and Foo, 2000; Chung, 2003) in mixture (3:1) [Fr. 4-9] (42.7 mg), chlorogenic acid (Scarpati et al., 1957; Han et al., 2006), and shikimic acid in mixture (2:1) [Fr. 10-68] (98.0 mg; Xiao et al., 2008). A second column chromatography was, later, performed on the assembly of [Fr.10-68] deriving from this first separation using, this time, as eluting system, a mixture of chloroform/methanol at different concentrations also raising the polarity of the solution during the run in order to elute the most polar compounds which, otherwise, would be left attached to the silica gel. The initial concentration of the mixture was CHCl_3_/MeOH 85:15 v/v and then it was passed respectively to 8:2 v/v, 7:3 v/v, and lastly to 6:4 v/v. From this run, shikimic acid [Fr. 4-25] (40.3 mg; Xiao et al., 2008) and chlorogenic acid [Fr. 39-100] (66.8 mg; Scarpati et al., 1957; Han et al., 2006) were better separated each other. The isolated compounds were identified by comparison of experimental NMR spectra with literature data and/or direct comparison with pure compounds available in our laboratory. NMR spectra were recorded on a Varian Mercury 300 MHz and/or on a Bruker Avance II 400 MHz instrument using CDCl_3_, CD_3_OD, or D_2_O as deuterated solvents; the chemical shift was expressed in ppm from TMS. MS spectra were performed on a Q-TOF MICRO spectrometer (Micromass, now Waters, Manchester, UK) equipped with an ESI source, that was operated in the negative and/or positive ion mode. The flow rate of sample infusion was 10 μL/min with 100 acquisitions per spectrum. Data were analyzed using the MassLynx software developed by Waters.

1,2,3,5-tetrahydroxyxanthone: ^1^H NMR (300 MHz, CD_3_OD) δ: 7.92 (1H, d, *J* = 7.8 Hz, H-8), 7.63 (1H, m, H-7), 7.44 (1H, d, *J* = 7.8 Hz, H-6), 6.33 (1H, s, H-4). ESI-MS: *m/z* [M-H]^−^ 259.07.

isoquercitrin: ^1^H NMR (300 MHz, CD_3_OD) δ: 7.89 (1H, s, H-2′), 7.67 (1H, d, *J* = 8.0 Hz, H-6′), 6.82 (1H, d, *J* = 8.0 Hz, H-5′), 6.46 (1H, br s, H-8), 6.17 (1H, br s, H-6), 5.15 (1H, d, *J* = 7.6 Hz, H-1″), 3.96 (1H, d, *J* = 12.0 Hz, H-6α″), 3.74 (1H, dd *J* = 12.0, 5.8 Hz, H-6β″). ESI-MS: *m/z* [M+Na]^+^ 486.87.

7-*O*-glucosyl luteolin: ^1^H NMR (300 MHz, CD_3_OD) δ: 7.39 (1H, br s, H-2′), 7.58 (1H, br s, H-5′), 7.07 (1H, d, *J* = 8.5 Hz, H-6′), 6.87 (1H, s, H-6), 6.46 (1H, br s, H-8), 5.34 (1H, d, *J* = 5.4 Hz, H-1″). ESI-MS: *m/z* [M+K]^+^ 486.95.

chlorogenic acid: ^1^H NMR (CD_3_OD a 300 MHz) δ: 7.57 (1H, d, *J* = 15.9 Hz, H-10), 7.03 (1H, br s, H-12), 6.93 (2H, m, H-15 to H-16), 6.28 (1H, d, *J* = 15.9 Hz, H-9), 5.35 (1H, m, H-3), 4.37 (1H, m, H-4), 3.67 (1H, m, H-5), 2.37-1.86 (4H, m, H-2 to H-6). ESI-MS: *m/z* [M+Na]^+^ 376.98; *m/z* [M-H]^−^ 353.21.

shikimic acid: ^1^H NMR (300 MHz, CD_3_OD), δ: 6.63 (1H, m, H-2), 4.32 (1H, br t, *J* = 3.9 Hz, H-3), 3.94 (1H, ddd, *J* = 7.6, 6.3, 5.2, Hz, H-5), 3.59 (1H, dd, *J* = 7.6, 3.9 Hz, H-4), 2.77 (1H, dd, *J* = 17.7, 5.2 Hz, H-6α), 2.18 (1H, dd, *J* = 17.7, 6.3, Hz H-6β). ESI-MS: *m/z* [M-H]^−^173.11.

### Determination of the antioxidant activity

#### 1,1-diphenyl-2-picrylhydrazyl (DPPH) radical-scavenging activity

The antiradical activity was determined by the DPPH radical-scavenging method (Peterson et al., 2002). Each sample was mixed with 900 μL of 100 mM Tris-HCl buffer, pH 7.4, and then added to 1 mL of 0.5 mM DPPH in methanol (250 μM in the reaction mixture). The control sample was prepared using methanol. Trolox was employed as a standard antioxidant to examine the radical-scavenging activities. Absorbances of the mixtures were measured at 517 nm. The activity was calculated as IC_50_. All tests and analyses were run in triplicate and averaged.

#### ß-carotene/linoleic acid assay

In this assay, the antioxidant capacity was determined by measuring the inhibition of the volatile organic compounds and the conjugated diene hydroperoxides arising from linoleic acid oxidation (Dapkevicius et al., 1998). A stock solution of β-carotene/linoleic acid mixture was prepared as follows: 0.5 mg of β-carotene was dissolved in 1 mL of chloroform, then 25 μL of linoleic acid and 200 mg of Tween 40 were added. Then, 100 mL distilled water saturated with oxygen (30 min, 100 mL/min) was added; 2.5 mL of this reaction mixture were dispensed into test tubes and 100 μL portions of variable concentrations of the samples were added; the emulsion system was incubated for up to 48 h at 37°C. The same procedure was repeated with the synthetic antioxidant butylated hydroxytoluene (BHT) and Trolox as positive controls, and the blank. After incubation, absorbances of the mixtures were measured at 490 nm. The activity was calculated as IC_50_. All tests and analyses were run in triplicate and averaged.

#### Hypochlorous acid scavenging (HOCl)

The amount of HOCl was measured by the chlorination of taurine (Weiss et al., 1982). One hundred microliters of sodium hypochlorite (600 mM) were added to 100 μL of taurine (150 mM) and 100 μL of variable concentrations of sample in PBS at pH 7.4. Absorbance was measured at 350 nm after the addition of 100 μL of 2 M potassium iodide. The activity was calculated as IC_50_. Trolox was used as the positive control. All tests and analyses were run in triplicate and averaged.

#### Cytotoxic activity

A375 (human malignant melanoma cells) and MDA-MB 231 cells (human breast adenocarcinoma cells) were cultured in Dulbecco's Modified Eagle's Medium (DMEM) with 2 mM L-glutamine, 100 IU/mL penicillin, 100 μg/mL streptomycin, and supplemented with 10% heat-inactivated fetal bovine serum (HI-FBS). HCT116 cells (human colon carcinoma cells), were cultured in RPMI1640 medium with 2 mM L-glutamine, 100 IU/mL penicillin, 100 μg/mL streptomycin, and supplemented with 10% HI-FBS. Cells medium and solutions were from PAA Laboratories GmbH, Austria. Cells were cultured in a humidified atmosphere at 37° C in presence of 5% CO_2_. The MTT assay was used as a relative measure of cell viability. Cell-viability assays were carried out as described (Quassinti et al., 2013). Briefly, cells were seeded at the density of 2 × 10^4^ cells/mL. After 24 h, samples were exposed to different concentrations of *H. androsaemum* fruits methanolic extracts (1.56–200 μg/mL). Cells were incubated for 72 h in a humidified atmosphere of 5% CO_2_ at 37°C. Cisplatin (Sigma) was used as the positive control. At the end of incubation, each well received 10 μL of 3-(4,5-dimethyl-2-thiazolyl)-2,5-diphenyl-2H-tetrazoliumbromide (MTT) (5 mg/mL in phosphate-buffered saline, PBS) and the plates were incubated for 4 h at 37°C. The extent of MTT reduction was measured spectrophotometrically at 540 nm using a Titertek Multiscan microElisa (Labsystems, FI-Helsinki). Experiments were conducted in triplicate. Cytotoxicity is expressed as the concentration of berries extract inhibiting cell growth by 50% (IC_50_). The IC_50_ values were determined with GraphPad Prism 4 computer program (GraphPad Software, S. Diego, CA, USA).

#### Immunomodulatory activity

Peripheral blood mononuclear cell (PBMC) proliferation assay was performed by flow cytometry on pig lymphocytes isolated from fresh heparinized blood samples (20 mL/pig).

The blood donors (Danish pigs, weighing 80–90 kg) were conventionally reared in a hilly area of Umbria (Rustici Farm, Parco del Subasio, Assisi, Italy) and the same were not subjected to none experimental protocol. For this reason this study is exempt from the ethics committee approval (DL 2014/26, Art. 2, comma 1,f). However, the blood samplings have been collected (only one time) with the farmer ‘s consent, handling the animals in accordance with the recommendations of the Directive 2010/63/EU of the European Parliament and of the Council of the European Union for the protection of animals used for scientific purposes. The number of live lymphocytes, suspended in complete RPMI-1640 medium (Euroclone^Ⓡ^) that contained 10% heat-inactivated pig serum, L-glutammine (2 mM; Euroclone^Ⓡ^), penicillin (100 U/mL; Biochrom^AG^, Berlin), and streptomycin (100 μg/mL; Biochrom^AG^, Berlin), was determined using a counting chamber and a trypan blue dye exclusion procedure. The final concentration of live cells was adjusted to 2 × 10^6^/mL in complete medium and 100 μL of suspension/well (2 × 10^5^ live cells) were dispensed in flat bottom 96-well tissue culture plates (Becton Dickinson, Lincoln Park, NJ). For the measurement of cell proliferation, the PBMC were prestained with carboxyfluorescein diacetate succinimydyl ester (CFSE) cell tracer (BioLegend, San Diego, CA) and cultured for 5 days at 37°C in 5% CO_2_. Proliferation stimuli were 1 μg/mL of pokeweed mitogen (PWM, it stimulates the B lymphocyte only in the presence of T cells; Sigma-Aldrich) or 1.2 μg/mL of phytohemagglutinin (PHA, a polyclonal T-cell activator; Biochrom^AG^, Berlin) in presence or absence of different dilutions of methanolic extract from red berries. The first assay was conducted analysing three different concentrations of red berries methanolic extract, starting from its IC_50_ higher dosage (i.e., 20, 10, and 6 μg/mL, respectively). The second assay was conducted further analyzing lower concentrations of *H. androsaemum* extract (i.e., 1.2, 0.8, and 0.4 μg/mL, respectively). Each culture condition was repeated in triplicate. The fruit extract was initially diluted 1:100 (v/v) in ethanol, and further dilutions were made in HBSS (Gibco^Ⓡ^, Life Technologies Italia). Finally, medium volume was adjusted to 200 μL/well. A negative control was represented by PBMC cultured without any mitogen/activator (CTR), so that the base proliferation could be estimated (Liu et al., 1996). With each cell division, the intensity of CFSE staining is reduced of a half and lymphocyte proliferation is calculated as frequency of CFSE^low^ cells within gated cell population compared to intensity of parent population. Flow cytometry analyses were performed on a standard FACSCalibur™ flow cytometer (Becton Dickinson, Mountain View, CA) operated by the CELLQuestPro™ software. Within a tight lymphocyte gate, 10,000 cells were acquired and the data were saved in the list mode. Cells from each culture condition were pooled and analyzed together. The percentage of increased proliferation vs. the basal values (PI) were calculated by the following formula:

$$\begin{array}{l} \left(\text{B}-\text{A}\right)∕{\text{A}}^{*}100 \end{array}$$

where, B is represented by the percentage of proliferation obtained by cells stimulated with the mitogen/activator ± fruit extract (the CTR-B was stimulated only with fruit extract), whereas A is represented by the percentage of proliferation obtained by cells stimulated or not (CTR) with the mitogen/activator, without fruit extract.

## Results and discussion

### Analysis of polar constituents in *H. androsaemum* fruit and infusions

From the analysis of *H. androsaemum* fruits methanolic extracts performed with the first method developed, we identified and quantified simultaneously 11 compounds, mainly polyphenols, i.e., shikimic acid, gallic acid, catechin hydrate, epicatechin, *p*-coumaric acid, *trans*-resveratrol, caffeic acid, *trans*-ferulic acid, chlorogenic acid, neochlorogenic acid, and 3,5-di-O-caffeoylquinic acid, meanwhile with the second method, we identified and quantified simultaneously seven compounds among flavonoids naphtodianthrones and phloroglucinols, i.e., rutin, quercetin, quercitrin, isoquercitrin, hyperoside, hypericin, and hyperforin.

In Table 2, the quantitative determination of the 18 analyzed compounds in the seven *H. androsaemum* fruit samples and in two infusions obtained from red and black “berry-like” capsules is reported. Shikimic acid, chlorogenic acid, rutin, and hyperoside, were present in all samples analyzed meanwhile neochlorogenic acid and isoquercetrin were not found only in black sample 5. Catechin was present only in the three red samples analyzed and in one black fruit sample (7), while epicatechin was present in the three red fruits but also in one black sample (6), even if in a very low concentration. Shikimic acid was found at high levels in all samples, ranging from 0.805 to 12.799 mg/g dry weight. It was found in higher concentration in red fruits (8.187–12.799 mg/g) than in black ones (0.805–5.988 mg/g). This compound is the biosynthetic precursor of aromatic amino acids and phenolic compounds and is endowed with important biological properties such as antiviral, anti-inflammatory, antiplatelet aggregation, and prevention of brain damage after ischemia. Shikimic acid was never reported in *H. androsaemum* leaves in previous studies.

**Table 2:** **Quantitative determination of the analyzed compounds in six ***H. androsaemum*** fruit samples (mg/g dry weight) and in two infusions (mg/L) obtained from red and black “berry-like” capsules; relative standard deviations were in a range from 0.10 to 5.88 (***n*** = 3)**.

**Constituent (mg/g dry fruit)**	**Fruits (mg/g)**							**Infusion (mg/L)**	
	**Red**			**Black**					
	**1**	**2**	**3**	**4**	**5**	**6**	**7**	**Red (2)**	**Black (5)**
Shikimic acid	12.799	10.203	8.187	1.235	1.182	0.805	5.988	208.0	29.5
Gallic acid	nd	nd	nd	nd	0.044	nd	0.032	nd	4.6
Caffeic acid	nd	nd	nd	nd	nd	nd	nd	nd	nd
Cumaric acid	nd	nd	nd	nd	nd	nd	nd	nd	nd
Ferulic acid	nd	nd	nd	nd	nd	nd	0.123	15.0	nd
Chlorogenic acid	14.553	7.035	6.811	2.029	0.099	0.441	0.887	422.0	8.4
Neochlorogenic acid	6.587	0.662	0.122	0.312	nd	0.008	0.035	nd	0.6
3,5-dicaffeoylquinic acid	0.208	0.340	nd	nd	nd	nd	nd	nd	nd
Catechin	0.114	0.063	0.083	nd	nd	nd	0.068	nd	23.2
Epicatechin	0.521	0.680	0.300	nd	nd	0.001	nd	25.0	nd
Rutin	0.233	0.662	0.008	0.039	0.014	0.023	0.011	27.0	nd
Hyperoside	0.662	0.053	0.004	0.247	0.015	0.012	0.029	21.6	nd
Isoquercetrin	0.179	0.106	0.004	0.078	nd	0.007	0.063	8.76	nd
Quercitrin	nd	0.113	0.017	nd	0.005	0.012	0.132	9.38	3.57
Quercetin	nd	nd	nd	nd	nd	nd	nd	nd	nd
Resveratrol	nd	nd	nd	nd	nd	nd	nd	nd	nd
Hyperforin	nd	nd	nd	nd	nd	nd	nd	nd	nd
Hypericin	nd	nd	nd	nd	nd	nd	nd	nd	nd

*nd, not detected*.

Chlorogenic acid was the second most abundant fruit constituent, ranging from 0.099 to 14.553 mg/g. The lowest amount was detected in black fruit (0.099 mg/g in sample 5) and the highest amount in the three red fruit samples, i.e., 14.553 mg/g in sample 1, 7.035 mg/g in sample 2, and 6.811 mg/g in sample 3. The high levels of chlorogenic acid found in this study are of great interest for a potential application of the berries of *H. androsaemum* as a functional food. Chlorogenic acid is a hydroxycinnamic acid derivative widespread in plants, fruits and vegetables, among which coffee beans are the main source. This compound has attracted attention of nutritionists because it has been proven to inhibit carcinogenesis, to protect against oxidative stress, to improve the glucose metabolism, to reduce the risk of cardiovascular disease, and to exhibit anti-obesity effects (Cho et al., 2010). Moreover, its abundance may also confirm the traditional uses of the plant as a wound healing agent (Allen and Hatfield, 2004). In fact, Chen et al. (2013) have demonstrated that topical application of chlorogenic acid can accelerate the process of excision wound healing by its ability to increase collagen synthesis through up-regulation of key players such as tumor necrosis factor-α and transforming growth factor-β1 in different phases of wound healing as well as by its antioxidant potential. Among the other compounds of chlorogenic acid family, neochlorogenic acid was quite abundant, ranging from 0.008 to 6.587 mg/g in the seven fruit samples. The lowest amount was detected in black fruits (sample 6) and the highest in red fruits (sample 1). Instead, 3,5-dicaffeoyilquinic acid was only present in red fruits (0.208 mg/g in sample 1 and 0.340 mg/g in sample 2). The concentrations of the caffeoylquinic acids found in the berries extracts were slightly lower with respect to those reported for water infusions of leaves (Valentão et al., 2004). In particular the latter showed neochlorogenic acid more abundant than chlorogenic acid, and high level of quercetin, which instead was missing in berries.

As mentioned above, catechin was present only in the three red fruits analyzed, ranging from 0.066 mg/g in sample 2 to 0.114 mg/g in sample 1, and in one sample (7) of black fruits (0.068 mg/g). Epicatechin amounts ranged from 0.001 mg/g in black fruits (sample 6) to 0.680 mg/g in red fruits (sample 2). The highest levels of these two flavanols, which are believed to possess strong antioxidant activity, were comparable with those previously found in the leaves (Dopico-Garcıa et al., 2011).

Among flavonoids, rutin was abundant in red fruits, ranging from 0.008 in sample 3 to 0.662 mg/g in sample 2. On the contrary, it was present in low amount in black fruits, ranging from 0.011 in sample 7 to 0.039 mg/g in sample 4. Although high, these levels were lower than those reported in other *Hypericum* species such as *H. perforatum* (4.25–9.23 mg/g), and *H. hyssopifolium* (12.42 mg/g), both growing in central Italy (Sagratini et al., 2008). This flavonoid was not reported in leaves of *H. androsaemum*.

Also hyperoside and isoquercitrin were present at high levels in red fruits, especially in sample 1 (0.662 and 0.179 mg/g, respectively), and in sample 2 (0.053 and 0.106 mg/g, respectively). These metabolites were already detected in leaves, although in trace amounts (Valentão et al., 2002). By the way, their levels were lower with respect to those found in other species belonging to the sect. *Androsaemum* like *H. grandifolium* (1.7 mg/g) and *H. hircinum* (1.3 mg/g; Bonkanka et al., 2008; Sagratini et al., 2008).

Quercitrin was found in two red fruit samples, such as sample 2 (0.113 mg/g) and sample 3 (0.017 mg/g), and in three black fruit samples, such as sample 5 (0.005 mg/g), sample 6 (0.012 mg/g), and sample 7 (0.132 mg/g). Also this compound was never detected in *H. androsaemum* leaves.

The HPLC-DAD method employed did not allow to detect both hypericin and hyperforin in the *H. androsaemum* fruits. The absence of hypericin, and of naphtodianthrones in general, is supported by the lack of black nodules which are the secretory structures storing naphtodianthrones. As a matter of fact, hypericin was never detected in the whole plant as well (Kitanov, 2001). Regarding hyperforin, other studies confirmed its absence from *H. androsaemum* (Aziz et al., 2006).

The performed analysis clearly showed that red fruits contained more constituents and in higher amount with respect to black ones, although some differences among samples according to the geographical origin were evidenced (Table 2). Samples 1, 2, and 3 of red fruits contained 9, 10, and 9 of the investigated analytes, respectively. Among black fruits, sample 7 was the richer with seven compounds. Overall, the fruits of *H. androsaemum* showed to be a rich source of shikimic acid. Moreover, they were richer in chlorogenic acid and poorer in neo-chlorogenic acid with respect to leaves previously analyzed (Valentão et al., 2004), while they lack quercetin which instead was found as the most abundant flavonoid in leaves (Dopico-Garcıa et al., 2011). On the other hand, fruits showed levels of the flavanols catechin and epicathechin comparable to those of leaves (Dopico-Garcıa et al., 2011).

From the analyses of the two infusions obtained from red and black “berry-like” capsules, it emerged that the former contained a high number of the investigated analytes (8 out of the 18 monitored) and at a quite high level (Table 2). In fact, the infusion obtained from red fruits was rich in chlorogenic acid (422 mg/L) and shikimic acid (208 mg/L), followed by rutin (27 mg/L), epicatechin (25 mg/L), and hyperoside (21.6 mg/L), while ferulic acid, quercitrin, and isoquercitrin were found at low concentrations (≤15 mg/L). On the other hand, the infusions obtained from black berries contained a lower number of the investigated analytes (6 out of 18 compounds) and in a very low level (<30 mg/L). In black berries infusion shikimic acid was the most abundant compound with a concentration of 29.5 mg/L. Surprisingly, catechin but not epicatechin was detected (23.2 mg/L). Overall, the analysis of infusions supported those performed on methanolic extracts and confirmed that red berries of *H. androsaemum* are to be preferred to black ones as a source of shikimic acid and phenolic compounds. The level of chlorogenic acid found in the infusion from red berries is particular interesting, since the concentration (422 mg/L) was comparable to that obtained in a cup of espresso coffee (Caprioli et al., 2013), in which it ranged from 394.9 to 555.8 mg/L, depending on the different ways of preparation. Also when compared with berry fruits such as blueberries, blackberries, and raspberries, tutsan fruits showed higher levels of chlorogenic acids (Clifford, 1999).

### Determination of the fruit pigment

**Figure 1:**
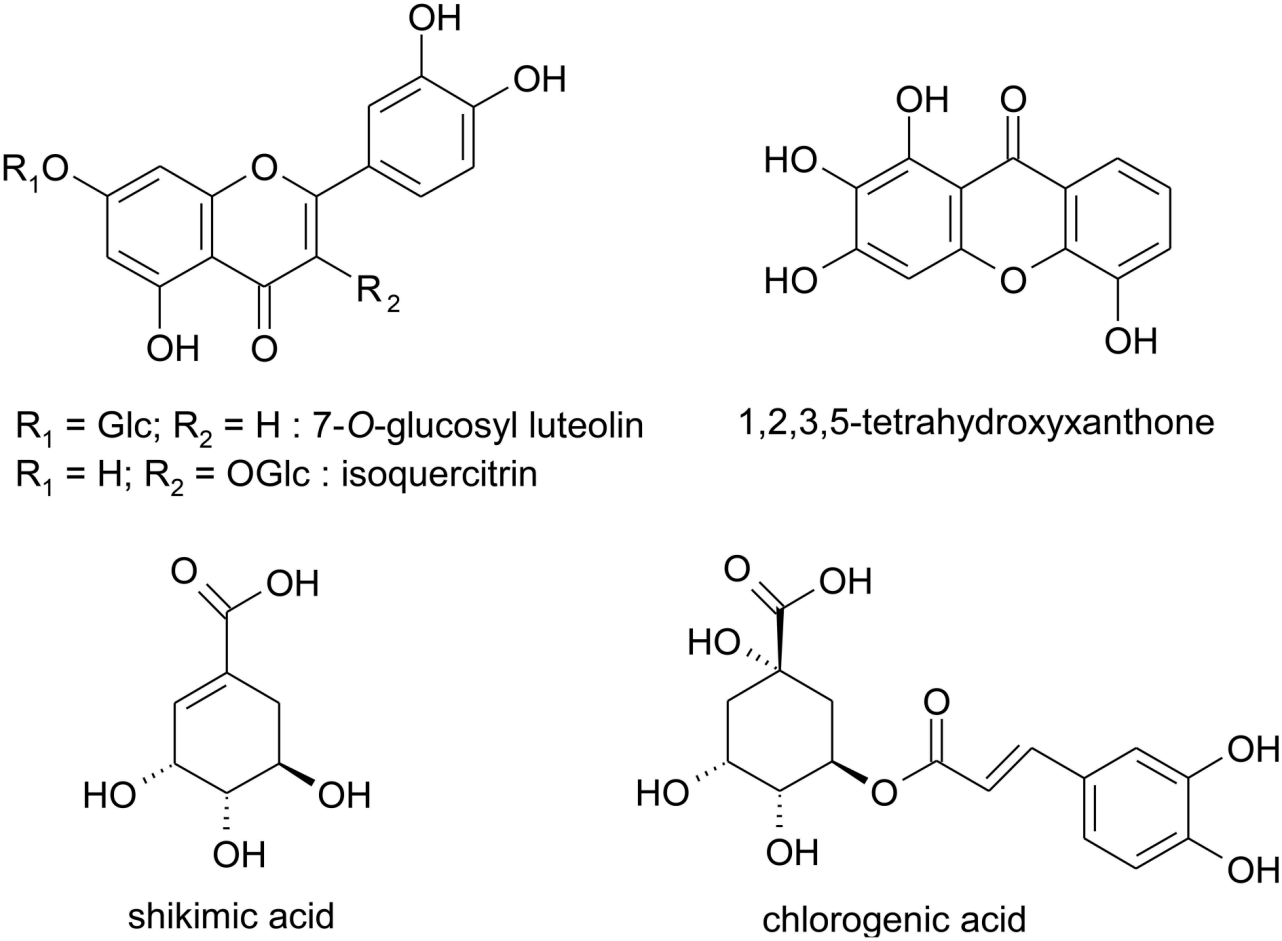
**Structures of polar compounds isolated from the red berries of ***Hypericum androsaemum*****.

The chromatographic separation of the ethanolic extract obtained from the red berries of *H. androsaemum* confirmed that chlorogenic and shikimic acids were the most abundant polar compounds. On the other hand, among the minor components we evidenced several molecules with aromatic structure such as glycosidic flavonoids (i.e., isoquercitrin and 7-*O*-glucosyl luteolin) and a xanthone never reported before in *Hypericum* species. The latter was identified as 1,2,3,5-tetrahydroxyxanthone (Figure 1), and its presence in *H. androsaemum* could be of taxonomic interest. This compound was previously found in *Polygala karensium* (Trong Tuan et al., 2012), a species belonging to Polygalaceae family. Previously, hydroxy and methoxy substituted xanthones were isolated from roots of *H. androsaemum* (Nielsen and Arends, 1979), while in calli and suspended cells cultures of the same species 1,3,5,6 and 1,3,6,7 oxygenated xanthones (Dias et al., 2000), along with prenylated xanthone aglycones and their glucosides were characterized (Schmidt et al., 2000). Therefore, the occurrence of this xanthone in the genus *Hypericum* is remarkable. Although xanthones are less abundant in the genus *Hypericum* and more in general in nature in comparison with other phenolic compounds, they showed several biological properties, such as strong and selective inhibition of MAO-A, *in vitro* toxicity, *in vivo* antitumor activity, as well as anti-inflammatory, antibacterial, and antifungal activities (Demirkiran, 2007).

The presence of mixture of all these aromatic compounds (i.e., flavonoids and xanthone) in the fruit pericarp, may contribute to the observed color of the fleshy capsule and extracts of *H. androsaemum*.

### Determination of total phenolics and flavonoids content, and of antioxidant capacity of *H. androsaemum* berries

The methanolic extracts from berries of *H. androsaemum* in almost all cases exhibited a significant high content in phenolics ranging from 1448 mg gallic acid equivalent (GAE)/kg in black fruits (sample 5) to 8530 mg GAE/kg in red berries (sample 2; Table 3). Much higher was the phenol content in tea infusions, with values of 8744 mg GAE/kg for black berries and, more important, of 18145 mg GAE/kg for red berries. The total phenols and the total flavonoids were “extracted” better with an infusion procedure than with a methanolic extraction. Therefore, the red fruits showed higher amounts of phenols and flavonoids than black ones, i.e., ripening lowered the phenolic content.

**Table 3:** **Antioxidant activity, total phenols, and total flavonoids in methanolic extracts and infusions of red and black berries of ***H. androsaemum*****.

**Sample**	**Color**	**DPPH μg/mL**	**Linoleic Test μg/mL**	**Hypochlorous acid μg/mL**	**Total Phenols mg gallic acid Eq/kg fw**	**Total Flavonoids mg rutin Eq/kg fw**
**METHANOLIC EXTRACTS**						
2	Red	27.7 ± 2.5bc^*^	11.7 ± 1.1bc	45.5 ± 4.1e	8530 ± 773b	1748 ± 158d
3	Red	29.5 ± 2.5cd	21.3 ± 1.8f	9.5 ± 0.8a	2158 ± 186a	1289 ± 111c
4	Black	33.9 ± 2.3cd	9.6 ± 0.7bc	20.1 ± 1.4bc	3114 ± 215a	676 ± 46ab
5	Black	32.1 ± 2.8cd	13.0 ± 1.1cd	16.9 ± 1.5b	1448 ± 126a	367 ± 32a
7	Black	36.5 ± 3.0d	23.7 ± 2.0f	23.3 ± 1.9c	2317 ± 194a	960 ± 80bc
**INFUSIONS**						
2	Red	21.4 ± 1.8b	7.9 ± 0.7b	37.0 ± 3.1d	18145 ± 1510c	2463 ± 205e
5	Black	52.8 ± 4.5e	17.1 ± 1.5e	10.6 ± 0.9a	8744 ± 738b	1140 ± 96c
**POSITIVE CONTROL**						
Trolox		6.3 ± 0.6a	16.6 ± 1.5de	10.6 ± 1.0a		
BHT			3.4 ± 0.3a			

* *Values within a column for each sample having different letters are significantly different from each other using Tukey's LSD test (p < 0.05)*.

On the basis of classification of fruits and vegetables depending on the total phenolic content, namely high (>2000 mg GAE/kg), medium (1000–2000 mg GAE/kg) and low (<1000 mg GAE/kg), the examined samples of berries fruits took place mainly in the first group. Interestingly, in almost all cases the total phenolic content of tutsan berries was higher than that of strawberry (1127 mg GAE/kg), mandarin (1161 mg GAE/kg), blueberry (2196 mg GAE/kg), and sour cherry (2560 mg GAE/kg; Dragović-Uzelac et al., 2009).

The chemical complexity of the extracts or infusions, often mixtures of many compounds with differences in functional groups, polarity, and chemical behavior, could lead to scattered results, depending on the antioxidant test employed. Therefore, an approach with multiple assays in screening work is highly advisable. The *H. androsaemum* berries were screened for their possible antioxidant activity by three non-enzymatic test systems: 1,1-diphenyl-2-picrylhydrazyl (DPPH) radical-scavenging, β-carotene/linoleic acid assay, and hypochlorous acid scavenging (HOCl). All methanolic extracts and tea infusions of berry-like fruits of *H. androsaemum* showed a significant radical scavenging activity with IC_50_ values in the range 21.4–52.8 μg/mL (Table 3). Methanolic extracts displayed similar inhibition on DPPH. Compared with activity of Trolox, the methanolic extracts showed an activity only 4.4–5.8 lower than that of the positive control. Water infusion from red berries was by far the most active with an IC_50_ value of 21.4 μg/mL, only 3.4 times higher than that of Trolox. This activity seemed to be related to the higher content of total phenolics (18145 mg GA Eq/kg).

The antioxidant activity of tutsan berries was evaluated by the β-carotene-linoleic acid test, using BHT as positive control. For the methanolic extracts we obtained IC_50_ values in the range 9.6–23.7 μg/mL which were 2.8–6.9 times higher than that of BHT. This activity did not depend on the ripening rate, but appeared to be correlated to the different geographic origin of the samples. Instead, water infusions displayed the same kind of inhibition seen on DPPH, with those prepared with red berries (IC_50_ value of 7.9 μg/mL) being more active than those made with black berries (IC_50_ value of 17.1 μg/mL). Opposite situation was found in the hypochlorous acid test where methanolic extracts showed in almost all cases a more potent scavenging activity (IC_50_ values in the range 9.5–45.5 μg/mL) with respect to the infusion made with red berries (IC_50_ value of 37.0 μg/mL). On the contrary, the black berries tea exhibited a good activity with an IC_50_ value of 10.6 μg/mL (Table 3).

*H. androsaemum* is a medicinal plant species containing many polyphenolic compounds, namely flavonoids, and phenolic acids and traditionally employed in the preparation of an infusion used for its diuretic and hepatoprotective activities (Valentão et al., 2004). A previous report investigated the ability of *H. androsaemum* leaf infusion to act as a scavenger of reactive oxygen species (superoxide radical, hydroxyl radical, and hypochlorous acid). The tested infusion mainly exhibited a potent scavenging effect on superoxide radicals, although a non-competitive inhibitory effect on xanthine oxidase was also observed. The infusion also acted as a moderate scavenger of hydroxyl radicals and hypochlorous acid (Valentão et al., 2002). Also our results supported the use of *H. androsaemum* in folk medicine to prepare teas with diuretic and antihepatotoxic activities.

### Cytotoxic activity

The cytotoxic activity of methanolic extracts of *H. androsaemum* fruits were evaluated on tumor cell lines by MTT assay. Three human cell lines, a malignant melanoma cell line (A375), a breast adenocarcinoma cell line (MDA-MB 231), and colon carcinoma cell line (HCT116), were treated with different concentrations of extracts for 72 h. As shown in Table 4, extracts were active against all three tumor cell lines tested and induced a concentration-dependent inhibitory effect in the dilution range 1.56–200 μg/mL. Results showed that the highest activity was observed on HCT116 cell line, with a IC_50_ value of 8.40 μg/mL for black berries extract, while red berries extract resulted less active on all cell lines tested (IC_50_ values in the range 19.40–32.29 μg/mL). Analysis of secondary metabolites put in evidence the presence of polyphenols, especially shikimic acid, chlorogenic acid, neochlorogenic acid, and 3,5-di-O-caffeoylquinic acid as the main components of fruit methanolic extracts. Chlorogenic acid is reported cytotoxic on human oral tumor cell lines but at high concentrations (HSG, HSC-2, and HGF; IC_50_ values of 1.4, 1.3, and 2.3 mM, respectively; Jiang et al., 2000). The same low cytotoxic activity was reported for shikimic acid on CHO, 3T3, and NRK cell lines (Ngomuo and Jones, 1996). 3,5-Dicaffeoylquinate inhibited proliferation in a dose-dependent manner as detected by MTT assays using Hela cells (Hu et al., 2014). Also flavonoids present in fruit extracts as catechin, epicatechin, and rutin were able to inhibit MDA-MB 231 and HCT116 proliferation at concentrations higher than 100 μM (Hayes et al., 2006; Li et al., 2009). Quercitrin has antiproliferative and apoptotic effect on colon cancer cells (Cincin et al., 2015). Given the cytotoxic properties reported in literature for xanthones (Bennet and Lee, 1989; Vieira and Kijjoa, 2005; Demirkiran, 2007), 1,2,3,5-tetrahydroxyxanthone may also contribute to the final effect observed on tumor cells. Our data confirm the properties of *H. androsaemum* to inhibit the proliferation of colon tumor cell lines as reported by Xavier et al. (2012). Aqueous extract of *H. androsaemum* leaves, tested on two human colon cancer-derived cell lines, HCT15 and CO115, inhibits proliferation and induced apoptosis through MAP kinases and PI3K/Akt pathway. The phenolic components of the *H. androsaemum* fruits methanolic extracts could explain the cytotoxic activity on tumor cells. Although polyphenols are generally recognized as antioxidants, they also act as prooxidants inducing DNA degradation in the presence of metal ions such as copper. Copper-dependent prooxidant mechanism of action of polyphenols accounts for their observed chemopreventive properties, as also for their preferential cytotoxicity toward cancer cells (Khan et al., 2012).

**Table 4:** **Cytotoxicity on tumor cells of the methanolic extracts from ***H. androsaemum*** berries**.

**Extracts**	**Cell line (IC**_**50**_ μ**g/mL)**^**a**^		
	**A375^b^**	**MDA-MB 231^c^**	**HCT116^d^**
Black berries	19.31	12.88	8.40
95% C.I.^e^	18.77-19.86	12.33-13.45	8.07-8.74
Red berries	32.29	30.05	19.40
95% C.I.^e^	31.45-33.16	26.77-33.74	18.43-20.42
**POSITIVE CONTROL**			
Cisplatin	0.38	2.57	2.42
95% C.I.^e^	0.31-0.47	2.23−3.05	2.08−2.91

a *IC_50_ = The concentration of compound that affords a 50% reduction in cell growth (after 72 h of incubation)*.

b *Human malignant melanoma cell line*.

c *Human breast adenocarcinoma cell line*.

d *Human colon carcinoma cell line*.

e *Confidence interval*.

In accordance with the US NCI criterion of anticancer activities (Boik, 2001), the *H. androsaemum* berries extracts may be considered as a potential source of cytotoxic drug, as they showed IC_50_ values below 20 μg/mL.

### Immunomodulatory activity

**Figure 2:**
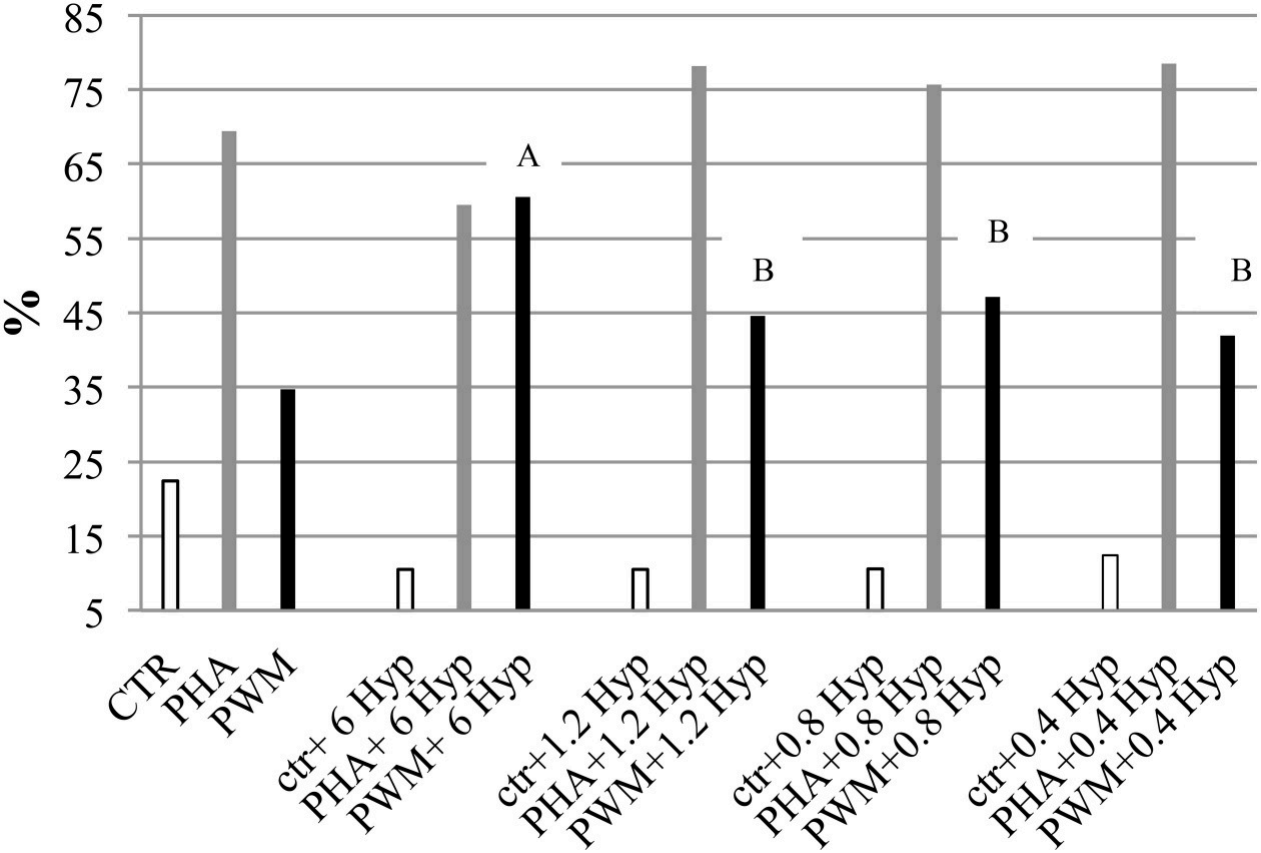
**Pig peripheral blood mononuclear cells (PBMC) proliferation assay monitored by CFSE labeling in response to ***in vitro*** stimulation with different dosage of ***H. androsaemum*** methanolic fruit extracts (expressed in μg/ml) ± mitogen or activator**. Data are shown as means (standard error: 6.27) and it was observed a significant effect of interaction between mitogen/activator and fruit extract dosages (*P* < 0.006). ^A, B^: different letters denote the significant differences of increased proliferation vs. the basal values (CTR, PHA, and PWM cells cultured without fruit extracts, but only mitogen/activator) obtained by adding different concentrations of *H*. *androsaemum* methanolic fruit extracts, *P* < 0.005.

The immunomodulatory activity of *H. androsaemum* red berries was evaluated by the *in vitro* proliferation assay of pig's PBMC which were stimulated with decreasing dilutions of red berries extract. In the first experiment, the two higher concentrations of red berries methanolic extracts tested (20 and 10 μg/mL, respectively), after 5 days of culture, revealed a cytotoxic effect that destroyed all the cells. Whereas, as shown in Figure 2, an immunodulatory effect started to be seen by using concentrations from 6 to 0.4 μg/mL.

Indeed, at the latter concentrations, while the addition of fruit extract to the unstimulated cells continued to elicit an inhibitory/cytotoxic (apoptosis has not been investigated in this study) effect on their proliferation, on the cells activated by PHA and PWM, the fruit extract elicited an increased proliferation response.

The dosage of 6 μg/mL of fruit extract gave the highest proliferative response on cells co-stimulated by PWM, possibly B cells. However, by decreasing the dosage of *H. androsaemum* fruits, the cells co-stimulated by PHA too increased their proliferation responses vs. control ones (CTR + PHA). The polyclonal activator of T cells (PHA) induced the higher PBMC proliferation response when combined with the lower fruit extract dosages investigated. Although, the treatment (different fruit extract concentrations) did not show a significant effect on the mean percentages resulted by the proliferative assay, a significant interaction between fruit extract dosage and mitogen/activator was observed (*P* < 0.01). Indeed, the PI of cells stimulated with PWM and 6 μg/mL of *H. androsaemum* extract was significantly higher than those obtained by cells cultured with PWM and 1.2, 0.8, and 0.4 μg/mL of fruit extract, respectively.

At the dosage of 6 μg/mL, the methanolic extract of red berries seemed to induce an immune deviation from cellular immunity to humoral responses that has been already observed by other authors in mice treated with hydroalcoholic extract of *H. perforatum* (Abhtai Froushani et al., 2015). The observed results may be correlated with the high levels of shikimic acid found in red berries. Shikimic acid was found effective, even in low doses, in the modulation of leukocyte activity (Bertelli et al., 2008). This compound, either alone or in combination with quercitin, was able to modulate the release of IL-6 and IL-8 from PBMCs. Since xanthones are molecules capable to modulate anti-inflammatory and anti-bacterial activities (Bennet and Lee, 1989), also the presence of 1,2,3,5-tetrahydroxyxanthone in the fruits of *H. androsaemum* may be taken into account for the observed activity.

The possibility to modulate the immune responses represents a very important goal when situations of immunodeficiency should be activated or a selective immunosuppression has to be induced, for example, in autoimmune disorders.

## Conclusion

The present study represents a comprehensive phytochemical and biological investigation of fruits of *H. androsaemum*, a wild medicinal plant of the Mediterranean region. On the above, the balsamic period was identified as the plant produces red berries. The latter were proven to be rich in shikimic acid and in phenolic compounds, especially chlorogenic acids. Moreover, the pigment giving the red color to the fruit pericarp was structurally determined as 1,2,3,5-tetrahydroxyxanthone that is reported for the first time for *Hypericum* species. Hence, *H. androsaemum* berries can be a good source of this molecule with potential applications at industrial level. Overall, the high antioxidant potential of tutsan berries was demonstrated and could be achieved directly by consumption of infusions or by incorporating polar extracts in antioxidant formulations. The cytotoxicity of methanolic extracts on tumor cell lines, especially on colon carcinoma, make *H. androsaemum* berries a potential candidate as a functional food having beneficial effects against tumors correlated with a life style characterized by the so-called “western diet.” Data from immunomodulatory assay suggest that red berries of *H. androsaemum* may represent a promising and natural strategy to modulate the immune system, although the mechanisms of action remain to be clarified. The obtained results could explain the past and current usage of *H. androsaemum* as food and in folk medicine; also they may support its further uses in health and in nutrition as a functional food.

## Author contributions

GC, HPLC analysis; AA, immunomodulatory assay; DB, immunomodulatory assay; AB, column chromatography and NMR; MB, MTT assay; CF, column chromatography and NMR; RI, HPLC analysis; FP, plant collection; LQ, MTT assay; GS, HPLC analysis; BT, antioxidant experiments; AV, column chromatography and NMR; SV, HPLC analysis.

### Conflict of interest statement

The authors declare that the research was conducted in the absence of any commercial or financial relationships that could be construed as a potential conflict of interest.

## References

[B1] Abhtai Froushani S. M. Galee H. E. G. Khamisabadi M. Lotfallahzades B. (2015). Immunomudulatory effects of hydroalcoholic extract of *Hypericum perforatum*. Avicenna J. Phytomed. 5, 62–68. 25767758 PMC4352534

[B2] Allen D. A. Hatfield G. (2004). Medicinal Plants in Folk Tradition. An Ethnobotany of Britain and Ireland. Portland, OR: Timber Press, Inc.

[B3] Aziz N. Sauve R. J. Long D. Cherry M. (2006). Genetic and Phytochemical Diversity Assessment Among Eleven *Hypericum* Accessions via AFLP and HPLC Analyses. J. Herbs Spices Med. Plants 12, 97–105. 10.1300/J044v12n01_09

[B4] Bennet G. J. Lee H.-H. (1989). Xanthones from Guttiferae. Phytochemistry 28, 967–998. 10.1016/0031-9422(89)80170-0

[B5] Bertelli A. A. E. Mannari C. Santi S. Filippi C. Migliori M. Giovannini L. (2008). Immunomodulatory activity of shikimic acid and quercitin in comparison with oseltamivir (Tamiflu) in an “*in vitro*” model. J. Med. Virol. 80, 741–745. 10.1002/jmv.21072 18297698

[B6] Boik J. (2001). Natural Compounds in Cancer Therapy. Princeton, MN: Oregon Medical Press.

[B7] Bonkanka C. X. Smelcerovic A. Zuehlke S. Rabanal R. M. Spiteller M. Sánchez-Mateo C. C. (2008). HPLC–MS analysis of anti-oedematogenic activity of *Hypericum grandifolium* Choisy (Hypericaceae). Planta Med. 74, 719–725. 10.1055/s-2008-1074526 18484525

[B8] Caprioli G. Cortese M. Odello L. Ricciutelli M. Sagratini G. Tomassoni G. (2013). Importance of Espresso coffee machine parameters on the extraction of chlorogenic acids in a certified Italian Espresso by Using SPE-HPLC-DAD. J. Food Res. 2, 55–64. 10.5539/jfr.v2n3p55

[B9] Chen W.-C. Liou S.-S. Tzeng T.-F. Lee S.-L. Liu I.-M. (2013). Effect of topical application of chlorogenic acid on excision wound healing in rats. Planta Med. 79, 616–621. 10.1055/s-0032-1328364 23568627

[B10] Cho A.-S. Jeon S.-M. Kim M.-J. Yeo J. Seo K.-I. Choi M.-S.. (2010). Chlorogenic acid exhibits anti-obesity property and improves lipid metabolism in high-fat diet-induced-obese mice. Food Chem. Toxicol. 48, 937–943. 10.1016/j.fct.2010.01.003 20064576

[B11] Chung H. S. (2003). Inhibition of monamine oxidase by a flavone and its glycoside from *Ixeris dentata* Nakai. Nutr. Food 8, 141–144. 10.3746/jfn.2003.8.2.141

[B12] Cincin Z. B. Unlu M. Kiran B. Bireller E. S. Baran Y. Cakmakoglu B. (2015). Apoptotic effects of quercitrin on DLD-1 colon cancer cell line. Pathol. Oncol. Res. 21, 333–338. 10.1007/s12253-014-9825-3 25096395

[B13] Clifford M. N. (1999). Chlorogenic acids and other cinnamates – nature, occurrence and dietary burden. J. Sci. Food Agric. 79, 362–372.

[B14] Dapkevicius A. Venskutonis R. Van Beek T. A. Linssen P. H. (1998). Antioxidant activity of extracts obtained by different isolation procedures from some aromatic herbs grown in Lithuania. J. Sci. Food Agric. 77, 140–146.

[B15] Demirkiran O. (2007). Xanthones in *Hypericum*: synthesis and biological activities. Top. Heterocycl. Chem. 9, 139–178. 10.1007/7081_2007_079

[B16] Dias A. C. P. Seabra R. M. Andrade P. B. Ferreres F. Fernandes-Ferreira M. (2000). Xanthone biosynthesis and accumulation in calli and suspended cells of *Hypericum androsaemum*. Plant Sci. 150, 93–101. 10.1016/S0168-9452(99)00178-8

[B17] Dopico-Garcıa M. S. Castro-Lopez M. M. Lopez-Vilarino J. M. Gonzalez-Rodrıguez M. V. Valentao P. Andrade P. B. (2011). Natural extracts as potential source of antioxidants to stabilize polyolefins. J. Appl. Polym. Sci. 119, 3553–3559. 10.1002/app.33022

[B18] Dragović-Uzelac V. Bursać Kovačević D. Levaj B. Pedisić S. Mezak M. Tomljenoviæ A. (2009). Polyphenols and antioxidant capacity in fruits and vegetables common in the croatian diet. Agric. Conspectus Scientificus 74, 175–179.

[B19] Han J.-T. Bang M.-H. Chun O.-K. Kim D.-O. Lee C.-Y. Baek N.-I. (2004). Flavonol glycosides from the aerial parts of *Aceriphyllum rossii* and their antioxidant activities. Arch. Pharm. Res. 27, P390–P395. 10.1007/BF02980079 15180303

[B20] Han T. Li H. Zhang Q. Zheng H. Qin L. (2006). New thiazinediones and other components from *Xanthium strumarium*. Chem. Nat. Comp. 42, 567–570. 10.1007/s10600-006-0215-2

[B21] Hayes C. J. Whittaker B. P. Watson S. A. Grabowska A. M. (2006). Synthesis and preliminary anticancer activity studies of C4 and C8-modified derivatives of catechin gallate (CG) and epicatechin gallate (ECG). J. Org. Chem. 71, 9701–9712. 10.1021/jo061740e 17168588

[B22] Hu T. He X. W. Jiang J. G. (2014). Functional analyses on antioxidant, anti-inflammatory, and antiproliferative effects of extracts and compounds from *Ilex latifolia* Thunb., a Chinese bitter tea. J. Agric. Food Chem. 62, 8608–8615. 10.1021/jf501670v 25118953

[B23] Jiang Y. Kusama K. Satoh K. Takayama F. Watanabe S. Sakagami H. (2000). Induction of cytotoxicity by chlorogenic acid in human oral tumor cell lines. Phytomedicine 7, 483–491. 10.1016/S0944-7113(00)80034-3 11194177

[B24] Khan H. Y. Zubair H. Ullah M. F. Ahmad A. Hadi S. M. (2012). A prooxidant mechanism for the anticancer and chemopreventive properties of plant polyphenols. Curr. Drug Targets 13, 1738–1749. 10.2174/138945012804545560 23140285

[B25] Kitanov G. M. (2001). Hypericin and pseudohypericin in some Hypericum species. Biochem. Syst. Ecol. 29, 171–178. 10.1016/S0305-1978(00)00032-6 11106845

[B26] Lamaison J. L. Carnat A. (1990). Teneurs en principaux flavonoides des fleurset des feuilles de *Crataegus monogyna* Jacq. et de Crataegus laevigata (Poiret) DC. Pharm. Acta Helv. 65, 315–320.

[B27] Li L. Henry G. E. Seeram N. P. (2009). Identification and bioactivities of resveratrol oligomers and flavonoids from *Carex folliculata* seeds. J. Agric. Food Chem. 57, 7282–7287. 10.1021/jf901716j 19627089

[B28] Liu F. C. Coimbra R. Hoyt D. B. Junger W. G. (1996). Proliferation assays with human, rabbit, rat, and mouse lymphocytes. In Vitro Cell. Dev. Biol. Anim. 32, 420–523. 10.1007/BF02722976 8946221

[B29] Lu Y. Foo L. Y. (2000). Flavonoid and phenolic glycosides from *Salvia officinalis*. Phytochemistry, 55, 263–267. 10.1016/S0031-9422(00)00309-5 11142853

[B30] Manguro L. O. A. Ugi I. Lemmen P. Hermann R. (2003). Flavonol glycosides of *Warburgia ugandensis* leaves. Phytochemistry 64, 891–896. 10.1016/S0031-9422(03)00374-1 14559287

[B31] Mulinacci N. Ieri F. Giaccherini C. Innocenti M. Andrenelli L. Canova G.. (2008). Effect of cooking on the anthocyanins, phenolic acids, glycoalkaloids and resistant starch content in two pigmented cultivars of *Solanum tuberosum* L. J. Agric. Food Chem. 56, 11830–11837. 10.1021/jf801521e 19053373

[B32] Ngomuo A. J. Jones R. S. (1996). Cytotoxicity studies of quercetin, shikimate, cyclohexanecarboxylate and ptaquiloside. Vet. Hum. Toxicol. 38, 14–18. 8825742

[B33] Nielsen H. Arends P. (1979). Xanthone constituents of *Hypericum androsaemum*. J. Nat. Prod. 42, 301–304. 10.1021/np50003a012

[B34] Perrone R. De Rosa P. De Castro O. Colombo P. (2013). Leaf and stem anatomy in eight *Hypericum* species (Clusiaceae). Turk. J. Bot. 37, 847–858. 10.3906/bot-1206-22

[B35] Peterson D. M. Hahn M. J. Emmons C. L. (2002). Oat avenanthramides exhibit antioxidant activities *in vitro*. Food Chem. 79, 473–478. 10.1016/S0308-8146(02)00219-4

[B36] Phillips R. (1977). Wild Flowers of Britain. London, UK: Pan Books.

[B37] Quassinti L. Lupidi G. Maggi F. Sagratini G. Papa F. Vittori S.. (2013). Antioxidant and antiproliferative activity of *Hypericum hircinum* L. subsp. majus (Aiton) N. Robson essential oil. Nat. Prod. Res. 27, 865–868. 10.1080/14786419.2012.677044 22480321

[B38] Sagratini G. Ricciutelli M. Vittori S. Öztürk N. Öztürk Y. Maggi F. (2008). Phytochemical and antioxidant analysis of eight *Hypericum* taxa from Central Italy. Fitoterapia 79, 210–213. 10.1016/j.fitote.2007.11.011 18178326

[B39] Scarpati M. L. Oriente G. Panizzi L. (1957). Sui costituenti caffeici del carciofo. Ann. Chim. 47, 150.

[B40] Schmidt W. El-Mawla A. M. A. Wolfender J.-L. Hostettmann K. Beerhues L. (2000). Xanthones in cell cultures of *Hypericum androsaemum*. Planta Med. 66, 380–381. 10.1055/s-2000-8542 10865463

[B41] Singleton V. L. Rossi J. A. J. (1965). Colorimetry of total phenolics with phosphomolybdic-phosphotungstic acid reagents. Am. J. Enol. Vit. 16, 144–153.

[B42] Trong Tuan D. Thai Trung D. Phi Hung N. Eunhee K. Phuong T. T. Won K. O. (2012). Xanthones from *Polygala karensium* inhibit neuraminidases from influenza A viruses. Bioorg. Med. Chem. Lett. 22, 3688–3692. 10.1016/j.bmcl.2012.04.028 22552195

[B43] Valentão P. Carvalho M. Fernandes E. Carvalho F. Andrade P. B. Seabra R. M.. (2004). Protective activity of *Hypericum androsaemum* infusion against *tert*-butyl hydroperoxide-induced oxidative damage in isolated rat hepatocytes. J. Ethnopharmacol. 92, 79–84. 10.1016/j.jep.2004.02.004 15099852

[B44] Valentão P. Fernandes E. Carvalho F. Andrade P. B. Seabra R. M. Bastos M. D. L. (2002). Antioxidant Activity of *Hypericum androsaemum* infusion: scavenging activity against superoxide radical, hydroxyl radical and hypochlorous acid. Biol. Pharm. Bull. 25, 1320–1323. 10.1248/bpb.25.1320 12392087

[B45] Vieira L. M. M. Kijjoa A. (2005). Naturally-Occurring Xanthones: recent developments. Curr. Med. Chem. 12, 2413–2446. 10.2174/092986705774370682 16250871

[B46] Weiss S. J. Klein R. Slivka A. Wei M. J. (1982). Chlorination of Taurine by Human Neutrophils. J. Clin. Investig. 70, 598–607. 10.1172/JCI110652 6286728 PMC370261

[B47] Xavier C. P. Lima C. F. Fernandes-Ferreira M. Pereira-Wilson C. (2012). *Hypericum androsaemum* water extract inhibits proliferation in human colorectal cancer cells through effects on MAP kinases and PI3K/Akt pathway. Food Funct. 3, 844–852. 10.1039/c2fo10226a 22596086

[B48] Xiao C. Dai H. Liu H. Wang Y Tang H. (2008). Revealing the metabolomic variation of rosemary extracts using 1H NMR spectroscopy and multivariate data analysis. J. Agric. Food Chem. 56, 10142–10153. 10.1021/jf8016833 18800806

[B49] Zorzetto C. Sánchez-Mateo C. C. Rabanal R. M. Lupidi G. Petrelli D. Vitali L. A.. (2015). Phytochemical analysis and *in vitro* biological activity of three Hypericum specie from the Canary Islands (*Hypericum reflexum, Hypericum canariense* and *Hypericum grandifolium*). Fitoterapia 100, 95–109. 10.1016/j.fitote.2014.11.013 25464055

