# Supplementary material for: Polar Constituents and Biological Activity of the Berry-Like Fruits from Hypericum androsaemum L
Source: Front Plant Sci. 2016 Mar 1;7:232. doi: 10.3389/fpls.2016.00232 (PMC4771922; doi:10.3389/fpls.2016.00232)
Supplement: Supplementary file 1 [file Table1.docx]

**Table 1S.** Calibration data, including regression equation, correlation coefficient (*R*^2^); limits of detection (LODs; µg/ml), limits of quantitation (LOQs; µg/ml)

| Compound | Regression  Equation | *R*^2^ | LOD^a^ | LOQ^b^ |
| --- | --- | --- | --- | --- |
|  |  |  |  |  |
| Shikimic acid | y = 25.143x + 5.0336 | 0.9999 | 0.06 | 0.20 |
| Gallic acid | y = 23.74x + 2.9068 | 0.9999 | 0.10 | 0.40 |
| (+)-Catechin | y = 22.188x + 0.3341 | 1.0000 | 0.15 | 0.50 |
| (-)-Epicatechin | y = 21.262x + 2.3431 | 0.9999 | 0.15 | 0.50 |
| Caffeic acid | y = 36.258x + 4.6226 | 0.9999 | 0.01 | 0.05 |
| *p*-Coumaric acid | y = 41.603x – 0.992 | 0.999 | 0.03 | 0.1 |
| *trans*-Ferulic acid | y = 40.642x – 0.989 | 1.0000 | 0.01 | 0.05 |
| *trans*-Resveratrol | y = 23.388x + 0.391 | 0.999 | 0.1 | 0.5 |
| Chlorogenic acid | y = 17.1507x - 27.5322 | 0.9930 | 0.03 | 0.10 |
| 5-O-caffeoylquinic acid | y = 15.496x - 1.5918 | 0.9998 | 0.08 | 0.25 |
| 3,5-di-O-caffeoylquinic acid | y = 18.19.x - 2.2361 | 0.9998 | 0.1 | 0.3 |
| Rutin | y = 26.466x + 2.6982 | 0.9982 | 0.1 | 0.5 |
| Hyperoside | y = 32.426x - 20.7894 | 0.9993 | 0.1 | 0.5 |
| Isoquercitrin | y = 28.7013x - 5.2504 | 0.9994 | 0.1 | 0.5 |
| Quercitrin | y = 55.227x - 66.415 | 0.9996 | 0.1 | 0.5 |
| Quercetin | y = 71.829x - 71.103 | 0.9996 | 0.2 | 0.8 |
| Hyperforin | y = 7.7078x + 0.9519 | 0.9994 | 0.3 | 1 |
| Hypericin | y = 37.864x + 9.7612 | 0.9998 | 0.1 | 0.5 |

^a^LOD (limit of detection) = 3 *×* signal-to-noise (S/N) ratio. ^b^LOQ (limit of quantitation) = 10 *×* signal-to-noise (S/N) ratio.
